# Effects of health education during public health emergencies on the health literacy, emotion and coping style of Chinese junior middle school students: a randomized controlled trial

**DOI:** 10.1186/s12889-023-17108-2

**Published:** 2023-11-07

**Authors:** Zhou Wang, Tingye Gao, Guangjian Li, Gengjuan Dong, Yan Zhan, Bingqin Wang, Xugui Sun

**Affiliations:** 1 Psychiatry and Psychology Department, Changzhou Dean Hospital, Changzhou, Jiangsu Province China; 2 Public Health Management Department, Changzhou Dean Hospital, Changzhou, Jiangsu Province China; 3 Psychological Counseling Room, Changzhou Beijiao Junior Middle School, Changzhou, Jiangsu Province China; 4 Psychological Counseling Room, Changzhou Diaozhuang Junior Middle School, Changzhou, Jiangsu Province China; 5 Physical Education Teaching and Research Office, Wannan Medical College, Wuhu, Anhui Province China

**Keywords:** Public Health emergencies, Health Education, Junior Middle School Student, Health literacy, Emotion, Coping style

## Abstract

**Background:**

Schools are high incidence places for public health emergencies. Good health literacy helps students cope with public health emergencies. Overall, the health literacy of young students is relatively low. Health education can promote health literacy, but the health education related to public health emergencies for Chinese junior middle school students needs to be improved. To design and implement health education courses related to public health emergencies for junior middle school students and examine the impact on their health literacy, emotions, and coping styles.

**Methods:**

From March to December 2022, 724 students in Grade 7 and Grade 8 of two junior middle schools in Changzhou were randomly divided into a course group (n = 359) and a control group (n = 365). The course group received an age-appropriate health education course that addressed public health emergencies; there were 12 classes, one per week. The control group received general health education. One week before and after the courses, the two groups of students were assessed with the Adolescent Health Literacy Evaluation Scale under Public Health Emergencies (AHLES-PHE), the Depression Self-Rating Scale for Children (DSRSC), the Generalized Anxiety Disorder 7-item scale (GAD-7), and the Simplified Coping Style Questionnaire (SCSQ).

**Results:**

After the courses were completed, the scores of AHLES-PHE [156.0 (45.0,180.0) vs. 165.0 (54.0,180.0), *P* < 0. 05] in the course group increased significantly. The positive rate of DSRSC [81 (22.6%) vs. 57 (15.9%), *P* < 0.05] and GAD-7 [45 (12.5%) vs. 29 (8.1), *P* < 0.05]in the course group were significantly lower than those before courses. There was no significant difference in the above indices before and after courses in the control group (*P* > 0.05).

**Conclusion:**

This suggests that the health education courses related to public health emergencies designed in this study has an effect on improving health literacy, depression and anxiety in junior middle school students.

## Introduction

A public health emergency refers to a sudden outbreak of a major infectious disease, a mass disease of unknown cause, major food or occupational poisoning and other events that cause or may cause serious damage to public health [1]. Health literacy is an important factor affecting individual health [2], especially in the occurrence of public health emergencies, and individuals can use their health literacy to carry out effective protection to reduce harm [3]. In 2019, Parvaneh Vasli et al. [4] conducted a cross-sectional study among Iranian adolescents, suggesting that health beliefs and health literacy can predict the formation of healthy behaviors in adolescents with COVID-19. During the epidemic of COVID-19, it is necessary to take some effective measures to improve the health literacy of adolescents to improve their health behaviors. The World Health Organization defines health literacy as a cognitive and social skill that gives individuals the motivation and ability to acquire, understand and use information and to promote and maintain health [5]. Schools are places with a high incidence of public health emergencies [6], and the overall health literacy of adolescent students is low. Approximately 30% of youth and adolescents in the United States have a low health literacy level [6, 7]. According to a health literacy survey of Chinese residents in 2020, the health literacy level of adolescents is 27.4% [8], lower than that of young and middle-aged people. Adolescents lack the ability to respond to public health emergencies and are prone to physical and mental health problems. In 2021, The Lancet reported a significant increase in the incidence of depression and anxiety among adolescents worldwide during the COVID-19 pandemic [9]. A meta-analysis in 2021 showed that the prevalence of depression and anxiety among children and adolescents worldwide during the pandemic was 25.2% (95% CI, 21.2 -29.7%) and 20.5% (95% CI, 17.2 -24.4%), respectively [10]. A survey conducted in Beijing, China, during the COVID-19 in 2020 showed that the prevalence of depression and anxiety among adolescents in junior and senior high schools was 40.7% and 24.1% respectively, with a significantly higher rate [11]. Another study in China in 2020 showed that the prevalence of depressive symptoms, anxiety symptoms and depression and anxiety syndrome among Chinese high school students during COVID-19 was 43.7%, 37.4% and 31.3% respectively [12].

Health education is an important measure to promote health literacy. Health education means helping people acquire health knowledge, adopt healthy behavior skills and lifestyles, eliminate risk factors affecting health and promote physical and mental health through educational activities [13]. Junior middle school is a transitional stage from childhood to adolescence. The health education of junior middle school students in China is not perfect. On the one hand, Schools do not attach enough importance to health education, and generally focus on the study of cultural courses, with relatively little actual implementation of health education. Students have limited reserves of health knowledge. In 2021, Huijie Shen et al. [6] conducted a survey on teachers and other staff in 2988 primary and secondary schools in Beijing, Chongqing, and Yunnan, China. The results showed that 50% of schools regularly carried out health related courses, and teachers and other staff had omissions in their knowledge of public health emergencies. Yong Xu [14] proposed that in the context of public health emergencies, health education in Chinese schools has become a mere formality, with students lacking basic knowledge of infectious diseases, food poisoning prevention and control, low skills, poor psychological tolerance, and weak information discrimination ability. He suggested strengthening students’ health education and improving their health literacy. Lili Chen et al. [15] conducted a survey on infectious disease health literacy among 723 students from 5 primary and secondary schools in Fengtai District, Beijing from September 2018 to February 2019. The overall accuracy rates of students’ health knowledge, attitude, and behavior were 67.44%, 58.69%, and 63.51%, respectively. Further strengthening of infectious disease health education among students is needed. Zhen Sun et al. [16] conducted a survey on COVID-19 knowledge among 4531 students in three middle schools in Wuhan, China, in 2021. The results showed that the awareness rate of COVID-19 prevention and control knowledge was 71.46% and the possession rate of prevention and control behavior was 55.99%, which still needed to be improved. On the other hand, although research on the assessment of adolescent health literacy in public health emergencies has been a hot topic in recent years, there are few studies on health education for junior middle school students in public health emergencies. Yuancheng Li et al. [17] developed a health literacy assessment scale for infectious disease prevention among Chinese middle school students from the perspective of public health, Wen Zhang et al. [18] made a questionnaire on their ability to respond to public health emergencies to survey 7719 college students in six colleges and universities in Shandong Province, and Chengyi Jia et al. [19] surveyed 1949 college students in Shangri La City about their knowledge of the COVID-19, but these studies did not pay attention to the perspective of health education. Xin Qu et al. [20] conducted a health education study on 2870 students from 14 high schools in Tongzhou District, Beijing, China, in response to public health emergencies. The study suggested that intervention activities for students to respond to public health emergencies were effective, but this study did not focus on junior middle school students.

There are many cognitive behavioral theories related to health education, among which the classic theory is Knowledge-Attitude-Practice (KAP) [21], which divides the change in human behavior into three continuous processes: knowledge acquisition, belief generation and behavior formation. Knowledge is the basis of behavior change, and belief is the driving force of behavior change. Wang Gaoling’s team [22] adapted KAP as the theoretical model for a health literacy evaluation form for public health emergencies and used it to evaluate and educate the residents. Then, there is the ABC theory of emotional behavior [23]. A (activating event) is a stressful event, such as a public health emergency; B (belief) refers to knowledge belief or skills held by people about events, such as health literacy knowledge and skills; C (consequence) is emotional and behavioral consequences, such as emotional and psychological reactions and behaviors to cope with public health emergencies. In this study, health education courses were designed and carried out for junior middle school students through the combination of medicine and education regarding public health emergencies; and students’ health literacy, emotions and coping styles were evaluated before and after the courses, which was consistent with the ABC theoretical model.

## Methods

### Study design and participants

Convenience sampling was used to enroll seventh and eighth grade students from two junior middle schools in Changzhou, Jiangsu Province. All students were included except for those with serious physical and mental diseases. PASS11.0 software was used to estimate the overall probability of the sample size estimation method, with a test level of 0.85, allowable error of 0.05, and a two-sided test. According to the health literacy survey of Chinese residents in 2020, the health literacy level of adolescents was 27.4% [8], so the sample size would be 698. On a voluntary basis, there were 800 participants in 16 classes, and 724 valid questionnaires were collected for an effective rate of 90.5%. There were 542 students in grade 7, 182 students in grade 8, 402 boys and 322 girls, aged 12 to 15 years old with an average age of 13.2 ± 0.7. Due to the fact that students take classes and live as a unit, and in order to improve the representativeness of the sample, a stratified cluster random sampling method was adopted for grouping. Stratified by school and grade, with each class as a unit, half of the classes in each grade of each school were randomly selected as the course group, and the other half as the control group. Finally, the classes were randomly divided into two groups of eight classes: 359 in the course group and 365 in the control group. The study was approved by the Ethics Committee of Changzhou Dean Hospital. All the students and their parents signed informed consent, and the privacy of the questionnaire test and health education were protected.

### Procedures

From March to December 2022, in two junior middle schools, the project named “Healthy Together” was launched. The adolescent health education program was the joint effort of doctors and teachers who devised a questionnaire evaluation and administered the health education courses. The study adopted a group design, and the steps were as follows. (1) A baseline assessment was conducted. One week before the course, all students in two groups were assessed with assessment tools, including Adolescent Health Literacy Evaluation Scale under Public Health Emergencies (AHLES-PHE), Depression Self-Rating Scale for Children (DSRSC), Generalized Anxiety Disorder 7-item scale (GAD-7) and Simplified Coping Style Questionnaire (SCSQ). Students with serious problems were surveyed to inform teachers for follow-up and were given psychological counseling or referred to a hospital psychological clinic if necessary. (2) Health education courses were conducted. The course group received the health education course on public health emergencies, which was taught by hospital physicians in school-based curriculum. The control group received general health education, which was taught by teachers as party of their daily teaching activities. Physicians and teachers have an education level of bachelor’s degree or above and have received training from the research group before conducting courses. The student’s classroom participation rate is 100%. (3) A post-course assessment was conducted one week after the end of the course. The two groups of students were re-evaluated using the same questionnaires used at baseline. Quality control included conducting research-related training and course content guidance for physicians and teachers before the study began. Before the questionnaires were administered, students were given guidance about how to complete them to prevent invalid questionnaires. Interference factors were controlled by administering questionnaires during non-examination periods and preventing students from withdrawing from the project due to major changes.

### Health education course

The course group carried out the health education course, which was designed according to the characteristics of public health emergencies and was age-appropriate for junior middle school students. (1) The basic framework of the course design was based on the national policy outline of “Healthy China 2030”, the definition of health literacy and health education is taken as the entry point. According to the “Health Literacy of Chinese Citizens-Basic Knowledge and Skills (Trial)” issued by the National Health Commission, health literacy is divided into three aspects: basic health knowledge and concepts, healthy lifestyles and behaviors, basic skills and literacy, and six aspects of health: scientific outlook on health, prevention and treatment of infectious diseases, prevention and treatment of chronic diseases, safety and first aid, basic medical treatment and health information. These aspects of health reference information on public health emergencies and COVID-19 released by the World Health Organization and the National Health Commission and “Health Education Guidelines for Primary and Secondary Schools” issued by the Ministry of Education. (2) The course content considering the age characteristics of junior middle school students and vividly depicts public health emergencies, healthy lifestyles, information on health, mental health, general medicine and five other aspects of health. Each of the 12 classes included knowledge content and skill guidance. The health education courses had been evaluated by medical and educational experts for its educational validity and had been recognized by the school as part of its school-based curriculum. (3) Due to the pandemic, online and offline classes were carried out. (4) Courses were held in two junior middle schools from March to June 2022 and from September to December 2022, respectively. There are a total of 12 classes, one class per week, with each class lasting 40 min. The course was conducted during the school day but without detracting from other classes (Table 1). The control group conducted general health education, including general health knowledge, personal hygiene, and dietary safety, once a month for 40 min each time, completed in daily teaching activities.

**Table 1:** The content and implementation time of the health education course related to public health emergencies

Course Topics	Course Content	Concrete Content	Implementation Time
Knowledge and skills related to public health emergencies	Are we prepared for a public health emergency?	What is a public health emergency? What are the types of public health emergencies? How do we face the hazards of public health emergencies?	Week 1, 40 min
	I am a guardian of COVID-19 prevention and control	How much do we know about COVID-19? What should we do to prevent and control the epidemic? I’m the little guard against the epidemic – physical and mental resistance.	Week 2, 40 min
	Knowledge and protection of common infectious diseases	What are the types and manifestations of common infectious diseases? What are the transmission routes of tuberculosis, hepatitis B, hepatitis C and AIDS? How to stay away from the source of infection during the epidemic of infectious diseases?	Week 3, 40 min
	What should I do if there is a leakage of harmful toxins or radioactive substances (radiation)?	How to identify common hazard signs and harmful and toxic substances? How to protect against radiation and hazardous chemicals?	Week 4, 40 min
	Self-rescue in case of earthquake, fire, electric shock, and other disasters	How to handle earthquakes, fires, and electric shock, and the qualities and skills required for self-rescue.	Week 5, 40 min
Knowledge and skills related to healthy lifestyles	Things you didn’t know about life - Healthy teen life	Which lifestyle is beneficial to health? Which lifestyle is harmful to health? What should be paid attention to in healthy living for teenagers?	Week 6, 40 min
	Food is the top priority for the people, and food safety is greater than the top priority	What is the knowledge of food hygiene and harmful food additives? How to deal with food poisoning urgently?	Week 7, 40 min
	How much do you know about household medicines?	What is medication? How to configure a regular household medicine-chest? How to read drug instructions?	Week 8, 40 min
Information on health-related knowledge and skills	In the era of big data, how to eliminate pseudoscience and attain true information	What is big data? What are the big data under the epidemic? How to eliminate pseudoscience and attain true information?	Week 9, 40 min
Mental health-related knowledge and skills	The mysteries of psychology and adolescence	What is psychology and mental health? What are the psychological characteristics of adolescence? How to handle common psychological problems and interpersonal relationships in adolescents?	Week 10, 40 min
	Scientific psychological stress relief and emotional management	What is the knowledge of psychological stress testing and stress relief? How to manage common emotional distress, depression, and anxiety in adolescents? How to psychologically respond to public health emergencies.	Week 11, 40 min
General medicine related knowledge and skills	General medical knowledge and first aid operation experience	What are vital signs? How to handle injuries and fractures? Hand in hand teaching you how to perform cardiopulmonary resuscitation.	Week 12, 40 min

### Assessment tools

(1) The AHLES-PHE Chinese version was compiled by Zhou Wang et al. [24] based on the ABC emotional behavior theory, with A (activating event) refers to public health emergencies; B (belief) refers to the knowledge and beliefs of adolescents regarding public health emergencies; C (consequence) refers to the health behavior skills adopted by adolescents in response to public health emergencies, and referring to information on public health emergencies released by domestic and foreign authorities, 66 Articles on Health Literacy of Chinese Citizens and Guidelines for Health Education in Primary and Secondary Schools. It has good reliability, structural validity and content validity and can be used to evaluate the health literacy of junior middle school students in the context of public health emergencies. The Cronbach’s α coefficient and parity split-half coefficient of AHLES-PHE were 0.958 and 0.975, respectively. The 2-week test-retest reliability of AHLES-PHE was 0.753. AHLES-PHE with 45 items and 8 factors was obtained by exploratory factor analysis. The 8 factors could explain 61.3% of the total variation. The fitting indexes of the confirmatory factor analysis model were *χ*^2^/ df = 3.325, RMSEA = 0.052, GFI = 0.853, CFI = 0.912, TLI = 0.904, NFI = 0.880. Sensibility analysis of AHLES-PHE showed the Cronbach’s α ranged from 0.957 to 0.958. The scale consists of 45 items, including 8 dimensions: general knowledge of public health emergencies, classified knowledge of public health emergencies, knowledge related to healthy life, beliefs in coping with public health emergencies, epidemic prevention and control behavior, healthy life-related behavior, daily basic skills, first aid skills, etc. Each item is graded by four levels, with an assigned value of 1 to 4 points and a total score of 45 to 180 points. The higher the score, the higher the health literacy. This study was used to evaluate students’ health literacy from the perspective of public health emergencies. The Cronbach’s α coefficients of the scale before and after the course were 0.938 and 0.957, respectively. (2) The DSRSC was compiled by Birleson et al. [25]. according to the diagnostic criteria for depression, and the Chinese version was cited from relevant Chinese literature [26]. The Chinese version of DSRSC was translated by Chinese scholar Linyan Su et al. [26] and tested for reliability and validity in children and adolescents in 14 large and medium-sized cities in China. It has good reliability and validity and is suitable for the detection of depressive symptoms in children and adolescents in the past week. The Cronbach’s α coefficient of the scale is 0.73, with a split half reliability of 0.72 and a 2-week test-retest reliability of 0.65. The consistency between the item and the total score is between 0.2 and 0.60. The sensitivity and specificity of depressive symptoms were 86% and 82%, respectively, with a critical value of 15. There were 18 items in the scale, and each item was graded at three levels, with a value of 0 ~ 2 points. Items 3, 5, 6, 10, 14, 15, 17 and 18 were scored positively, while items 1, 2, 4, 7, 8, 9, 11, 12, 13 and 16 were scored inversely. The total score ranged from 0 to 36, and the higher the score was, the more obvious the depressive symptoms were. Considering the critical value, changes in scores alone cannot reflect the severity of depressive symptoms. Therefore, students were screened with a total score ≥ 15 (positive for depressive symptoms) in this study. Cronbach’s α coefficients of the scale before and after the course were 0.817 and 0.855, respectively. (3) GAD-7 is a short scale developed by Spitzer RL et al. [27] based on the American Manual for the Diagnosis and Statistics of Mental Disorders, used to assess anxiety symptoms in the past 2 weeks. The Chinese version is cited from Chinese literature [28, 29]. The Chinese version of GAD-7 was translated by Chinese scholar Xiaoyan He et al. [29] and has good reliability and validity in the diagnosis and screening of generalized anxiety scale in China. The Cronbach’s α coefficient of the scale is 0.898, and the 2-week test-retest reliability is 0.856. The consistency between the item and the total score is between 0.556 and 0.904. The sensitivity and specificity of using a critical value of 10 to determine anxiety symptoms were 86.2% and 95.5%, respectively, with a Kappa value of 0.825. There are 7 items in total, and each item is graded on four levels with values of 0 ~ 3 points. The total score ranges from 0 ~ 21 points, with a total score of 0 ~ 4 indicating no anxiety, 5 ~ 9 mild anxiety, 10 ~ 14 moderate anxiety, and 15 ~ 21 severe anxiety. Considering the critical value, the severity of anxiety symptoms cannot be fully reflected by the change in scores alone. Therefore, a total score ≥ 10 (positive anxiety symptoms) was used to screen students in this study. Cronbach’s α coefficients of the scale before and after the course were 0.879 and 0.905, respectively. (4) SCSQ Chinese version was created by a Chinese scholar named Yaning Xie [30] and can be used to evaluate the coping styles of adults and adolescents when they suffer setbacks. It has good reliability and validity with a Cronbach’s α coefficients of 0.90 for the entire scale and a 2-week test-retest reliability of 0.89. Factor analysis confirmed that the scale had a 2-factor structure. There are 20 items in total, and each item is graded on four levels, with scores of 0 ~ 3. Positive coping is evaluated in items 1 to 12, and the score ranges from 0 to 36. The higher the score is, the more positive the coping style. Negative coping includes items 13 to 20, with scores ranging from 0 to 24. The higher the score was, the more negative the coping style. This study was used to evaluate students’ style in coping with life events (such as public health emergencies). Cronbach’s α coefficients of the scale before and after the course were 0.802 and 0.813, respectively.

### Statistical methods

Epidata 3.1 was used for data entry, and SPSS 21.0 was used for statistical analysis. General demographic data statistics, age and other normally distributed measurement data were described by ($\bar x$±*s*) and compared using *t* tests. The count data were described by frequency statistics and compared by *χ*^*2*^ test. Before and after the course, the measurement data of nonnormal distribution were measured by *M* (*P*_*min*_, *P*_*max*_), nonparametric rank-sum test was used for comparison, Mann-Whitney U test with two independent samples was used for intergroup comparison, and paired sample Wilcoxon test was used for intragroup comparison. Statistical data were described by frequency statistics, and *χ*^*2*^ was used for comparisons between groups. Intra-group comparison was performed by McNemar’s test with paired samples. *P* < 0.05 was considered statistically significant.

## Results

### Demographic characteristics

Grade 7 and 8 students from two junior middle schools in Changzhou (n = 724) completed the entire study were randomly divided into a course group (n = 359) and a control group (n = 365). There was no statistical difference between the course group and the control group in terms of age, gender, grade, ethnicity, previous academic performance, family type, and parental education level. The two groups of students are balanced in demography characteristics (*P* > 0.05, Table 2).

**Table 2:** Baseline demographic characteristics of students stratified by the course and control group

	Course Group (n = 359)	Control Group (n = 365)	*t*/*χ*^2^	*P*
Age	13.1 ± 0.7 yrs	13.2 ± 0.7 yrs	-0.947	0.344
Gender				
Boys	195 (54.3)	207(56.7)	0.420	0.517
Girls	164 (45.7)	158(43.3)		
Grade level				
Seventh grade	265 (73.8)	277 (75.9)	0.414	0.520
Eighth grade	94 (26.2)	88 (24.1)		
Ethnicity				
Han nationality	354 (98.6)	363(99.5)	1.349	0.245
Ethnic minorities	5(1.4)	2(0.5)		
Previous academic performance				
Excellent	52 (14.5)	54 (14.8)	0.720	0.949
Good	121 (33.7)	115 (31.5)		
Average	108 (30.1)	109 (29.9)		
Poor	51 (14.2)	55 (15.1)		
Very Poor	27 (7.5)	32 (8.8)		
Family type				
One-child family	136 (37.9)	140 (38.3)	3.013	0.556
Multi-child family	135 (37.6)	128 (35.1)		
Three generations of the family	62 (17.3)	62 (17.0)		
Single-parent family	13 (3.6)	23 (6.3)		
Other families	13 (3.6)	12(3.3)		
Father’s education background				
Primary education and/or less	14 (3.9)	10 (2.7)	2.611	0.456
Junior middle school	104 (29.0)	122 (33.5)		
High school	79 (22.0)	83 (22.7)		
Bachelor level and/or higher	162 (45.1)	150 (41.1)		
Mother’s education Background				
Primary education and/or less	29 (8.1)	27(7.4)	1.246	0.742
Junior middle school	97(27.0)	108(29.6)		
High school	78(21.7)	85(23.3)		
Bachelor level and/or higher	155(43.2)	145(39.7)		

Note: Age and other normally distributed measurement data were described by Mean ± SD and compared using *t* tests. The count data were described by n (%) and compared by *χ*^2^ test

### Health literacy

Baseline AHLES-PHE scores in the two groups were comparable (*P* = 0.798, Table 3). After the course, there was no statistically significant difference between the course group and the control group (*P* = 0.153, Table 3). Upon comparing the AHLES-PHE scores of the course group before and after the course, a statistically significant increase (*P* = 0.014, Table 3) was observed. Conversely, the AHLES-PHE scores of the control group did not exhibit any significant difference before and after the course (*P* = 0.073, Table 3).

### Depression level

A comparison between the groups before the course revealed no statistically significant difference between the course group and the control group for positive rate of DSRSC (*P* = 0.404, Table 3). After the course, the course group was lower than the control group for positive rate of DSRSC, with a statistically significant difference (*P* = 0.012, Table 3). A comparison was made between the positive rate of DSRSC with the course group before and after the course, indicating statistically significant decrease in the positive rate after the course (*P* = 0.022, Table 3). However, the positive rate of DSRSC in the control group did not exhibit a significant difference before and after the course (*P* = 0.526, Table 3).

### Anxiety level

The baseline positive rate of GAD-7 was similar in the two group (*P* = 0.588, Table 3). After the course, there was no statistically significant difference between the course group and control group (*P* = 0.474, Table 3). Comparison within the group before and after the course, the positive rate of GAD-7 in the course group was lower after the course than before, with a statistically significant difference (*P* = 0.036, Table 3), while the positive rate of GAD-7 in the control group showed no statistically significant difference before and after the course (*P* = 0.794, Table 3).

### Coping style with setbacks

A comparison between the groups before the course revealed no statistically significant difference between the course group and the control group in the positive coping and negative coping scores of SCSQ (positive coping *P* = 0.259, negative coping P = 0.101, Table 3). After the course, there was no statistically significant difference between the course group and the control group (positive coping *P* = 0.092, negative coping *P* = 0.451, Table 3). In the course group, there was no statistically significant difference before or after the course (positive coping *P* = 0.285, negative coping *P* = 0.805, Table 3). In the control group, there was no statistically significant difference before or after the course (positive coping *P* = 0.100, negative coping *P* = 0.278, Table 3).

**Table 3:** Comparison of assessment results between the two groups

	Course Group (n = 359)		Control Group (n = 365)		Baseline		Course effect					
	Before course	After course	Before course	After course	^*a*^ *Z/χ* ^*2*^	^*a*^ *P*	^*b*^ *Z/χ* ^*2*^	^*b*^ *P*	^*c*^ *Z/χ* ^*2*^	^*c*^ *P*	^*d*^ *Z/χ* ^*2*^	^*d*^ *P*
AHLES-PHE scores	156 (45.0,180.0)	165 (54.0,180.0)	158 (75.0,180.0)	163 (45.0,180.0)	-0.256	0.798	-2.458	0.014	-1.793	0.073	-1.431	0.153
Positive DSRSC	81 (22.6)	57 (15.9)	92 (25.2)	85 (23.3)	0.695	0.404	5.676	0.022	0.581	0.526	6.303	0.012
Positive GAD-7	45 (12.5)	29 (8.1)	41 (11.2)	35 (9.6)	0.293	0.588	4.923	0.036	0.121	0.794	0.513	0.474
Positive SCSQ scores	3.1 (1.0,4.0)	3.2 (1.0,4.0)	3.1 (1.0,4.0)	3.0 (1.0,4.0)	-1.130	0.259	-1.070	0.285	-1.646	0.100	-1.685	0.092
Negative SCSQ scores	2.0 (1.0,4.0)	2.0 (1.0,4.0)	2.1 (1.0,4.0)	2.0 (1.0,4.0)	-1.642	0.101	-0.247	0.805	-1.085	0.278	-0.753	0.451

Note:

AHLES-PHE: Adolescent Health Literacy Evaluation Scale under Public Health Emergencies. AHLES-PHE Scores were nonnormal distribution measurement data, described by *M (P*_*min*_, *P*_*max*_*)*

DSRSC: Depression Self-Rating Scale for Children. Positive DSRSC were numbers of positive individuals screened by DSRSC, which was count data and described by *n* (%)

GAD-7: Generalized Anxiety Disorder 7-item scale. Positive GAD-7 were numbers of positive individuals screened by GAD-7, which was count data and described by *n* (%)

SCSQ: Simplified Coping Style Questionnaire. Positive SCSQ scores and Negative SCSQ scores were nonnormal distribution measurement data, described by *M (P*_*min*_, *P*_*max*_*)*

^a^ The baseline values were a comparison between course group and control group before the course. Two independent samples Mann-Whitney U test was used for intergroup comparison of nonnormal distribution measurement data, while intergroup comparison of count data was performed using *χ*^2^ test

^b^ Course effect for the course group between pre-course and post-course. Paired sample Wilcoxon test was used for intragroup comparison of nonnormal distribution measurement data, while intragroup comparison of count data was performed using McNemar’s test with paired samples

^c^ Course effect for the control group between pre-course and post-course. Paired sample Wilcoxon test was used for intragroup comparison of nonnormal distribution measurement data, while intragroup comparison of count data was performed using McNemar’s test with paired samples

^d^ Course effect for post-course between course group and control group. Two independent samples Mann-Whitney U test was used for intergroup comparison of nonnormal distribution measurement data, while intergroup comparison of count data was performed using χ ^2^ test

## Discussion

An examination of the impact of the course on students revealed the following. In terms of AHLES-PHE, the baseline level of the course group and the control group was balanced. The scores of the course group improved after the course compared with that before the course, and the difference was statistically significant, while the scores of the control group also improved after general health education, the difference was not statistically significant. The health education course designed in this study, which included public health emergencies, healthy life, health information, mental health, general medical knowledge and skills, had an effect on the improvement of students’ health literacy. The control group carried out general health education to help students understand general health knowledge and partially promote health literacy, so the scores of both the course group and the control group improved after the course, but there was no significant difference between the two groups. Xin Qu et al. [20] conducted a study on health education for 2870 students from 14 high schools in Tongzhou District, Beijing, China, in response to public health emergencies. The students were divided into urban intervention group, rural intervention group, and control group. The urban intervention group adopted activities such as essay writing, themed class lectures, knowledge promotion videos, and knowledge promotion materials for public health emergencies. The rural intervention group adopted special lectures, emergency drills, and knowledge promotion materials for public health emergencies. No activities were carried out in the control group. After intervention, the awareness rate of food poisoning (increased by 19.4% in urban areas and 13.7% in rural areas) and prevention of infectious diseases (increased by 19.1% in urban areas and 1.6% in rural areas) in urban and rural intervention groups significantly increased, while there was no significant change in the control group. Health education interventions for public health emergencies in schools were effective. Liping Yu et al. [31] conducted a survey on the health education needs of college students during public health emergencies and showed that 61.8% of college students hope that schools can provide comprehensive health education courses for students. Health education course is a convenient and effective way to improve health literacy. Schools should actively promote health education course to provide effective ways for students to understand public health emergencies and increase health knowledge.

In terms of DSRSC, the baseline levels of the course group and the control group were balanced. The positive rate of post-course depression in the course group was lower than that before the course, and the positive rate of post-course depression in the course group was lower than that in the control group, and the difference was statistically significant. The difference was not statistically significant before and after the general health education in the control group. The health education course designed in this study has content about public health emergencies and mental health, which has a guiding effect on the normal epidemic prevention and control, emotional management and interpersonal relationship handling of students in the course group, so it has an effect on the improvement of students’ depressive moods. Once depression occurs, more professional psychological counseling and education are needed to alleviate it. The control group only received general health education and lacked professional mental health education, so depression did not significantly improve. These results are similar to those of the study conducted by Heli Lu et al. [32] in China which conducted health management for adolescents with depression in remote counties. Health management based on the knowledge transformation model was provided in the intervention group, and routine health management was provided in the routine group. The results showed that the DSRSC scores of the intervention group was lower than that of the conventional group after intervention. Another intervention study also had similar results, but the intervention methods, grouping, and depression assessment scale were different. Qinghong Mao et al. [33] recruited 96 middle school students from various parts of China with depressive symptoms to participate in intervention research during the 2020 epidemic. The research subjects were divided into image intervention group (36 students), activity reminder (30 students), and blank group (30 students). The image intervention group were given positive image intervention of image script technique for two weeks, and the activity reminder group completed the task through daily activity reminders, The blank group did not receive any intervention, and the results showed the Beck Depression Scale score in the intervention group was significantly lower than the other two groups (F = 13.99, P < 0.001).

In terms of GAD-7, the baseline level of the study was balanced between the course group and the control group. The positive rate of anxiety in the course group after the course was lower than that before the course, and the difference was statistically significant, while the positive rate of anxiety in the control group also decreased after general health education, but the difference was not statistically significant. The health education course designed in this study, especially the content on mental health, has an effect on the improvement of students’ anxiety. The control group received general health education that helped students understand general health knowledge and coping skills and partly relieved anxiety. Therefore, after the course, the number of positive students in both the course group and the control group decreased, and there was no significant difference in the positive rate between the two groups. There is a meta-analysis that supports the conclusions of this study. Wendel Flora et al. [34] conducted a meta-analysis of anxiety intervention for children and adolescents during the prevalence of COVID-19. Seven randomized controlled trials were included. Intervention methods include professional and general interventions such as mental health education and physical exercise. The results showed that the anxiety of children and adolescents was reduced through intervention measures (Standardized Mean Difference (SMD) (95% CI): − 0.33 (− 0.59; − 0.06).

In terms of SCSQ, the results of this study showed that there were no statistically significant differences between the two groups before and after the course and within the group, indicating that there was no significant change in students’ coping styles as a result of the course or general health education. Possible reasons are as follows: First, junior middle school students faced much frustration and pressure, not only limited to public health emergencies. The course designed in this study was mainly about how to deal with public health emergencies. Second, Yaning Xie [30] suggests that although coping styles are related to emotional health, they have diversity and complexity. Different coping styles will have different outcomes for different environments and people. Positive and negative are relative, and positive emotions do not necessarily lead to positive coping styles, nor do positive coping styles necessarily lead to positive consequences. In this study, even though students’ negative emotions had been improved through health education courses, the change in coping styles might not be significant. Moreover, this study was evaluated one week after the course, and coping style was a relatively stable psychological state compared with emotional reaction, so perhaps the effect of the course had not been fully revealed. This is similar to a domestic school-based intervention study on suicide risk in junior middle school students [35]. After intervention, SCSQ scores of students in the intervention group and the control group did not change significantly.

This study was carried out during the epidemic period of COVID-19 in 2022. The research group and the school had made efforts to prevent and control the epidemic and prevent cross infection among two groups of students. During this study period, there were no reports of related cases of infection among the two groups of students. The measures taken were as follows: (1) In this study, the intervention group and control group were randomly selected based on the class as a unit, and the learning and life of two groups were relatively separate. (2) Adopt a combination of offline and online teaching methods and use online teaching when the epidemic was severe. (3) All research subjects signed informed consent forms, obtaining the consent and cooperation of students and parents. (4) During the epidemic, the school would also take routine prevention and control measures according to the epidemic prevention and control guidelines for middle school students, such as students wearing masks, washing hands frequently, measuring body temperature, regular nucleic acid testing, classroom disinfection and ventilation; If students had fever during temperature measurement, they would rest in the isolation room and take their temperature again. If their temperature was higher than normal, they would be taken back by their parents; If there were close contacts or suspected positive cases, students would be temporarily suspended from school, and parents would take students to the hospital for medical observation and treatment. These students would be withdrawn from the research project.

There are several limitations that suggest future research directions. The research subjects were junior middle school students from two middle schools. Thus, compared with teenagers in general, the findings for these subjects may not be generalizable. Future research can be carried out with larger sample sizes. The content of the course can be further adjusted, such as adding content on the ability to resist setbacks. Due to the pandemic, online and offline courses can be combined, and offline courses can be adopted when the pandemic eases. The follow-up time after the course of this study is slightly shorter, which could be extended in the future. Assessment tools, such as other health literacy assessment questionnaires, could be added to validate the effectiveness of the course.

## Conclusion

In conclusion, the health education course on public health emergencies designed and carried out in this study has an effect on the improvement of health literacy, emotions such as depression and anxiety in junior middle school students, while the impact on coping style is not obvious.

## Data Availability

The datasets generated and/or analyzed during the current study are not publicly available concerning students’ privacy and their own or guardian’s willingness but are available from the corresponding author Zhou Wang (13,685,292,630@163.com) on reasonable request.

## Abbreviations

AHLES-PHE: Adolescent Health Literacy Evaluation Scale Under Public Health Emergencies
DSRSC: Depression Self-rating Scale for Children
GAD-7: Generalized Anxiety Disorder 7-item Scale
SCSQ: Simplified Coping Style Questionnaire
